# Brain integrity is altered by hepatic *APOE* ε4 in humanized-liver mice

**DOI:** 10.1038/s41380-022-01548-0

**Published:** 2022-04-13

**Authors:** Andreas Giannisis, Kalicharan Patra, Anna K. Edlund, Lur Agirrezabala Nieto, Joan Benedicto-Gras, Simon Moussaud, Andrés de la Rosa, Daniel Twohig, Tore Bengtsson, Yuan Fu, Guojun Bu, Greg Bial, Lander Foquet, Christina Hammarstedt, Stephen Strom, Kristina Kannisto, Jacob Raber, Ewa Ellis, Henrietta M. Nielsen

**Affiliations:** 1 Department of Biochemistry and Biophysics, Stockholm University, Stockholm, 10691 Sweden; 2 Department of Molecular Biosciences, The Wenner-Gren Institute, Stockholm, 10691 Sweden; 3 Department of Neuroscience, Mayo Clinic College of Medicine, Jacksonville, FL 32224 USA; 4 Yecuris Corporation, Tualatin, OR 97062 USA; 5 Department of Laboratory Medicine (LABMED), Karolinska Institutet, Stockholm, 17177 Sweden; 6 Departments of Behavioral Neuroscience, Neurology, and Radiation Medicine, and Division of Neuroscience, ONPPRC, Oregon Health & Science University, Portland, OR 97239 USA; 7 Department of Clinical Science, Intervention and Technology, (CLINTEC), Division of Transplantation surgery, Karolinska Institutet, Huddinge, 14152 Sweden

**Keywords:** Neuroscience, Molecular biology

## Abstract

Liver-generated plasma apolipoprotein E (apoE) does not enter the brain but nonetheless correlates with Alzheimer’s disease (AD) risk and AD biomarker levels. Carriers of *APOE*ε4, the strongest genetic AD risk factor, exhibit lower plasma apoE and altered brain integrity already at mid-life versus non-*APOE*ε4 carriers. Whether altered plasma liver-derived apoE or specifically an *APOE*ε4 liver phenotype promotes neurodegeneration is unknown. Here we investigated the brains of *Fah−/−*, *Rag2−/−, Il2rg−/−* mice on the Non-Obese Diabetic (NOD) background (FRGN) with humanized-livers of an AD risk-associated *APOE* ε4/ε4 versus an *APOE* ε2/ε3 genotype. Reduced endogenous mouse apoE levels in the brains of *APOE* ε4/ε4 liver mice were accompanied by various changes in markers of synaptic integrity, neuroinflammation and insulin signaling. Plasma apoE4 levels were associated with unfavorable changes in several of the assessed markers. These results propose a previously unexplored role of the liver in the *APOE*ε4-associated risk of neurodegenerative disease.

## Introduction

*ΑPOE* in humans is polymorphic with the ε2, ε3 and ε4 alleles encoding the apolipoprotein E (apoE) isoforms apoE2, apoE3 and apoE4. Compared to ε3, ε4 increases the risk of developing Alzheimer’s disease (AD) and dementia with Lewy bodies (DLB) by up to 15- and 6-fold, respectively [1–3]. The underlying mechanisms were proposed to involve accumulation of brain amyloid-β plaque pathology even in cognitively healthy subjects [4, 5], cognitive injury prior to the development of plaque pathology as in mice expressing the human *APOE *ε4 and human *APP* with familial AD mutations [6], or altered brain insulin signaling [7, 8] and glucose metabolism [9, 10] resulting in brain insulin resistance and cerebral glucose hypometabolism [11–13].

Several studies have reported reduced plasma apoE levels in ε4-carriers [14, 15]. This reduction was evident in plasma only and not in cerebrospinal fluid (CSF), and specifically attributed to reduced apoE4 levels as shown in heterozygous individuals [16]. Also, the plasma composition of the two apoE isoforms in *APOE* heterozygous individuals differs from the apoE isoform composition in the CSF [16, 17]. Although low plasma apoE levels increases the risk of not only AD but all types of dementia [18], a peripheral phenotype based on altered plasma apoE levels with relevance to the brain under physiological or neurodegenerative conditions is controversial due to the inability of peripheral apoE to enter the central nervous system (CNS) [19]. However, we have described a correlation between an increased ratio of plasma apoE4 to apoE3 isoform levels, glucose hypometabolism specifically in the hippocampus, and reduced gray matter volume in several brain areas of relevance to AD [20]. Low plasma apoE levels were furthermore adversely linked to cognitive function and CSF markers of AD brain pathology [21]. We therefore hypothesize that a peripheral ε4 phenotype, despite the inability of apoE to enter the CNS [19] is related to the increased risk of developing neurodegenerative diseases. Importantly, plasma apoE levels per se may only serve as a promotor and/or surrogate marker of down-stream processes which in turn can translate into injury and pathological processes in the brain. To study and translate the results from such a scenario in rodent models to humans is difficult since mice inherently differ from humans in their lack of *APOE* polymorphism and by their dramatically different lipid metabolism [22]. The role of apoE4 in cognitive performance and AD has been assessed in mouse models [23], including models expressing *APOE* ε4 in brain on a murine *Apoe* deficient background, models expressing *APOE* ε4 by targeted *Apoe* replacement [24] and more recently also in models where specifically the rodent hepatic *Apoe* was replaced by human *ε*4 [25, 26]. The latter studies proposed a link between hepatic apoE4, an altered peripheral lipid metabolism, and synucleinopathy in brain. However, the described mouse models include human apoE in the context of a mouse liver metabolome and proteome. To study a potential relationship between human hepatic function, hepatic apoE, and processes promoting pathological processes in the brain, humanized-liver mice such as the humanized-liver *Fah−/−*, *Rag2−/−, Il2rg−/−* (FRG® -KO) mouse on the Non-Obese Diabetic (NOD) background (FRGN) which reproduces the human cholesterol lipoprotein profile [22, 27] may serve as a superior model.

In the current study, we assessed associations between a human *APOE* ε4/ε4 liver genotype and measures of synaptic integrity, brain insulin signaling and neuroinflammation in the cortex, hippocampus, the thalamus and the cerebellum. We compared FRGN mice with humanized-livers of an *APOE* ε4/ε4 to those of a non-ε4 genotype *APOE* ε2/ε3 in which the ε2 allele is known to be protective against AD [28].

## Materials and methods

### In vivo models

FRGN mice with humanized-livers were generated and kept in line with previous published protocols [29]. In brief, the mouse model was developed through knock-out of the *Fah*, *Rag2*, and *IL2rg* genes (FRG® -KO mouse) and then cross-bred with Non-Obese Diabetic (NOD) mice to generate the FRGN mouse [30, 31]. For the current study, a total of 18 mice were used. Seven mice (3 male and 4 female individuals) were transplanted with primary human hepatocytes derived from an *APOE* ε2/ε3 donor, and 11 mice (6 male and 5 female individuals) were transplanted with cells from two donors with an *APOE* ε4/ε4 genotype (for details see Supplementary Materials and Methods and Supplementary Table 1). The number of animals was restricted by the amount of primary human hepatocytes available at the time of transplantation and experiments were performed with the *APOE* genotypes blinded to the investigator. Mice were euthanized by exsanguination under anesthesia (isofluorane) at the age between 5–8 months, the average age was 7 months. Brains were carefully removed, divided into the right and left hemispheres, snap frozen and kept at −80 °C until processed. All institutional and national guidelines for the care and use of laboratory animals were followed and the herein described studies were conducted according to Karolinska Institutet guidelines and in agreement with the approved ethical protocol ID400 42-17.

*APOE*-targeted replacement (*APOE* TR) mice in which the murine *Apoe* gene locus is replaced with the human *APOE* ε3, or *APOE* ε4 gene [32] were obtained from Taconic Biosciences. Animals were housed under controlled temperature and lighting conditions, and were given free access to food and water. Three mice (two females and one male) of each genotype, *APOE* ε3 vs *APOE* ε4, were euthanized at 6–8 months of age. After transcardial perfusion with phosphate-buffered saline (PBS, pH 7.4), the brains were collected and divided along the sagittal plane, immediately snap-frozen in liquid nitrogen and further stored at −80 °C until further analysis. All animal procedures were approved by the Mayo Clinic Institutional Animal Care and Use Committee (IACUC) and were in accordance with the National Institutes of Health Guide for the Care and Use of Laboratory Animals. Mouse brain tissues were shipped to Sweden and imported with permission from the Swedish Board of Agriculture (6.7.18-7013/18), for biochemical analyses.

### Brain tissue dissection

The right hemispheres of the brains from 18 FRGN and 6 TR mice were thawed from −80 °C at room temperature in PBS (pH 7.4, 10 mM Na_2_HPO_4_, 1.8 mM KH_2_PO_4_ 137 mM NaCl, and 2.7 mM KCl), and dissected under the microscope (Nikon SMZ-U Zoom 1:10 Stereoscopic Microscope) to collect specifically the cortex, the hippocampus, the cerebellum and the thalamus. The dissected brain tissue areas were weighed and stored at −80 °C for further analysis. Cortex and hippocampus were dissected from 6 TR and 12 FRGN mice, whereas thalamus and cerebellum were dissected from 6 FRGN mice.

### Brain tissue fractionation

**Fig. 1:**
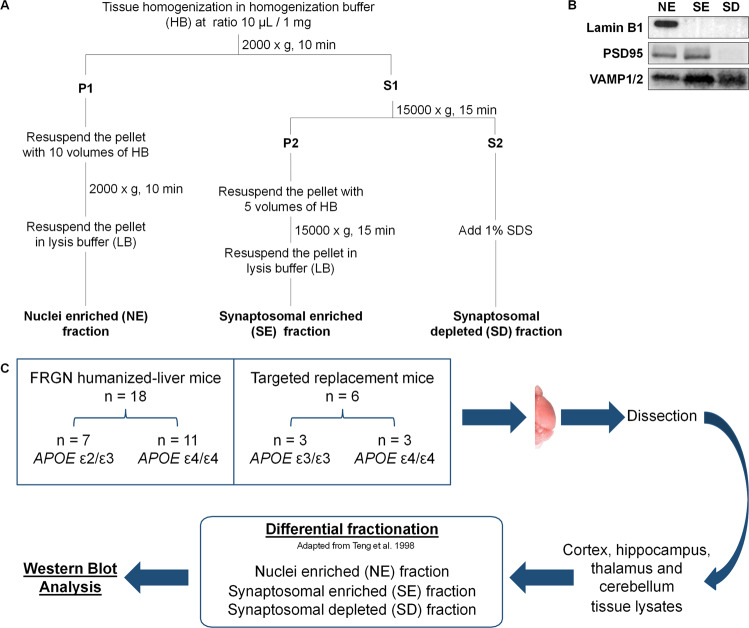
Study workflow. **A** Schematic illustration of the differential fractionation protocol employed for the preparation NE, SE and SD fractions of the dissected brain areas obtained from FRGN humanized-liver and *APOE* TR mice. **B** Validation of tissue fractionation efficiency. Lamin B1 was detected only in the NE fraction, while PSD95 was present only in the NE and SE fractions. Synaptobrevin isoforms 1 and 2 (VAMP1/2) was present in all the fractions. **C** Schematic experimental layout. The right brain hemispheres from *n* = 18 FRGN (whereof *APOΕ* ε2/ε3 *n* = 7 and *APOE* ε4/ε4 *n* = 11) and *n* = 6 TR (whereof *APOΕ* ε3 *n* = 3 and *APOE* ε4 *n* = 3) mice were utilized. The cortex and hippocampus were dissected from the right hemispheres of *n* = 6 TR mice (whereof *APOΕ* ε3 *n* = 3 and *APOE* ε4 *n* = 3) and *n* = 12 FRGN humanized-liver mice (whereof *APOΕ* ε2/ε3 *n* = 4 and *APOE* ε4/ε4 *n* = 8). Thalamus and cerebellum were dissected from the right hemispheres of *n* = 6 FRGN humanized-liver mice, (whereof *APOΕ* ε2/ε3 *n* = 3 and *APOE* ε4/ε4 *n* = 3).

Cortex, hippocampal, cerebellar and thalamic tissues were thawed on ice and, mixed with homogenization buffer (HB) (0.32 M sucrose, 5 mM HEPES, 2 mM EDTA, pH 7.4 and 1X protease and phosphatase inhibitors cocktail (Thermo Scientific)) in a ratio of 10 μL/1 mg tissue in glass tubes. The tissue was homogenized using a motor-driven glass teflon homogenizer (RW16 basic IKA®-WERKE) set at 700 RPM with 12 up and down slow strokes. The lysates were differentially fractionated to yield three separate fractions; nuclei enriched (NE), synaptosomal enriched (SE) and synaptosomal depleted fraction (SD), according to a previously published protocol [33] (Fig. 1A) (for details see Supplementary Materials and Methods). Each fraction was validated by identifying fraction-specific markers using SDS-PAGE under reducing conditions, followed by western blot (WB) analysis. Presence of lamin B1 in the NE fraction and not in the SE and SD fractions confirmed the purity of NE fraction, while absence of PSD95 from the SD fraction confirmed the separation of SE and SD fractions (Fig. 1B).

### Western blot analysis

Tissue fraction samples were mixed 3:1 with SDS-PAGE loading buffer (60 mM Tris-HCl, 2% SDS, 0.01% bromophenol blue and 10% glycerol, 2.5% β- mercaptoethanol), heated at 95 °C for 5 min and equal protein amounts were loaded into wells of 4–15% pre-cast polyacrylamide gels (Bio-Rad Tris-Glycine-TGX). The separated proteins were transferred to a polyvinylidene difluoride membrane (PVDF, Immobilon-P Millipore) using the Bio-Rad Trans-blot semi-dry system using 1X semi-dry transfer buffer (48 mM Tris base, 39 mM glycine, 0.0375% SDS and 20% methanol). The membranes were blocked either with 2% w/v non-fat dry milk powder, or 2% w/v bovine serum albumin (BSA) in tris-buffered saline (TBS, 20 mM Tris base and 150 mM NaCl) with 0.05% Tween-20 (TBS-T) for 1 h at room temperature, and then incubated with the corresponding primary antibody (Supplementary Table 2) diluted in blocking solution overnight at 4 °C. The detection of the studied proteins (Supplementary Table 3) was enabled by use of secondary antibodies conjugated with either horseradish peroxidase (HRP) (dilution 1:5000 in TBS-T) or with a fluorophore dye (800CW, or 680RD) (dilution 1:20000 in TBS-T). The visualization of HRP-secondary antibodies was performed by use of Advansta enhanced chemiluminescence (ECL) solutions (1:1) and the Bio-Rad ChemiDoc scanner. Membranes probed with fluorophore conjugated antibodies were visualized using the LI-COR Odyssey imaging platform. The freely available software Image J was used for the densitometric analysis of the WB-detected protein bands. Densities of the individual bands representative of various markers (Supplementary Table 3) were semi-quantified by employing the same size rectangular area for all marker-specific bands on individual membranes. The resulting arbitrary values were normalized against synaptobrevin isoforms 1 and 2 (VAMP1/2) as the expression levels of this protein exhibited the highest stability among all the assessed brain areas and tissue fractions, and did not differ between the investigated groups of mice. A schematic layout of the experimental strategy is visualized in Fig. 1C.

### Quantification of plasma apoE levels

Plasma samples from the FRGN mice were diluted in PBS containing 1% w/v non-fat dry milk powder and the levels of apoE were determined by use of a previously published sandwich enzyme linked immunosorbent assay (ELISA) [34] (see Supplementary Materials and Methods).

### Statistical analysis

The ELISA and WB-generated data were statistically analyzed using the JMP Pro statistical software version 14.0.0 (SAS Institute, NC, USA). Plasma apoE levels as well as densitometry-generated values of the studied proteins were assessed for normality using the Shapiro-Wilk test for goodness of fit. Variables that did not follow normal distribution were log-transformed and the distribution was re-assessed. Comparisons between variables that followed normal distribution either directly or after log transformation were performed using the Student’s *t* test. For non-normally distributed variables the non-parametric Wilcoxon signed-rank test was utilized. Linear regression analysis was used to assess associations between brain marker levels and plasma apoE4 before and after accounting for a potential interaction between plasma apoE4 levels and the corresponding hepatocyte *APOE* ε4/ε4 donor*: Model 1: plasma apoE4* versus Model 2: plasma apoE4* APOE ε4/ε4 donor. The results are reported as estimates with 95% confidence interval (CI).

## Results

### Plasma human apoE levels and endogenous mouse apoE in the FRGN humanized-liver mouse brain

**Fig. 2:**
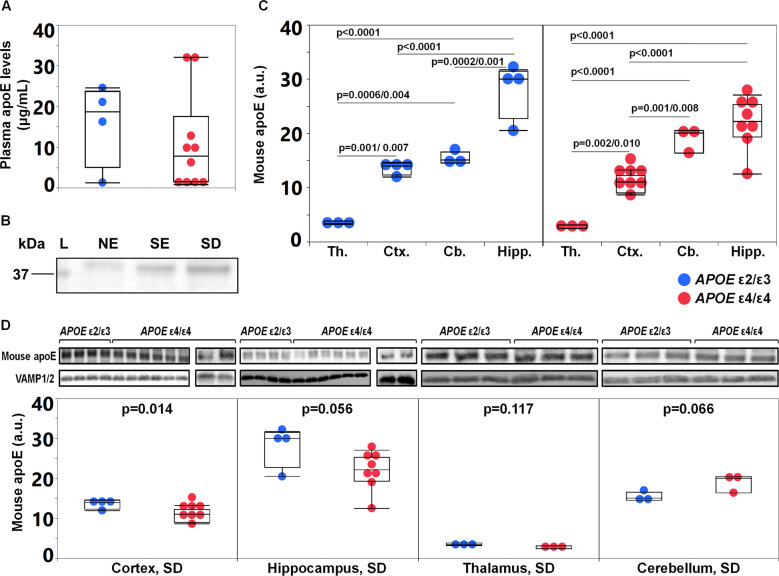
Plasma human apoE levels and endogenous mouse apoE in the FRGN mouse brain. **A** Plasma human apoE levels, assessed by ELISA *APOE* ε2/ε3 (*n* = 4) versus *APOE* ε4/ε4 (*n* = 10) FRGN humanized mice (*p* = 0.525, assessed by Wilcoxon signed-rank test). **B** Western blot image showing apoE immunoreactive bands in the NE, SE and SD cortical fractions. **C** Levels of brain apoE in the SD fraction prepared from thalamus (Th.), cortex (Ctx.), cerebellum (Cb.), and hippocampus (Hipp.) of *APOE* ε4/ε4 and *APOE* ε2/ε3 humanized-liver mice. Protein levels were normalized against synaptobrevin isoforms 1 and 2 (VAMP1/2). **D** Densitometric analyses of immunoreactive bands corresponding to endogenous mouse apoE after normalization against synaptobrevin isoforms 1 and 2 (VAMP1/2) in the SD fraction isolated from the cortex, hippocampus, thalamus and cerebellum from *APOE* ε2/ε3 and *APOE* ε4/ε4 FRGN humanized-liver mice. Data is shown as mean or median (minimum–maximum). Group comparisons were done using the Student’s *t* test (**C**, **D**), or Wilcoxon signed-rank test (**A**). See also Supplementary Fig. 1.

Plasma human apoE levels were quantified in a subset of the included animals; 4 mice with *APOE* ε2/ε3 livers and 10 mice with *APOE* ε4/ε4 livers (for specifics see Supplementary Table 1). The plasma concentrations of apoE were similar to those reported in humans and ranged between 1.3–24.6 μg/mL for *APOE* ε2/ε3 and 0.8–32.1 μg/mL for *APOE* ε4/ε4 mice (Fig. 2A). The plasma apoE4 levels generated in mice from two *APOE* ε4/ε4 donors were significantly different (Donor #2: *n* = 7 vs Donor #3: *n* = 3, *p* = 0.023, Wilcoxon signed-rank) however levels did not differ significantly between the two groups with livers of different *APOE* genotype (*p* = 0.525). Using the same anti-human apoE antibody (clone WUE4) as the one used as the capture antibody in the ELISA for western blotting, we were unable to detect human apoE in the brain tissues of the humanized-liver mice (data not shown).

In line with the notion that synapse dysfunction and failure intimately being related to neurodegeneration as in AD [35] we employed an adapted subcellular fractionation protocol [33] allowing enrichment of synaptic proteins as part of the synaptosome [36] yielding three different preparations; (nuclei enriched NE; synaptosomal enriched SE; synaptosomal depleted SD). Mouse endogenous apoE was detected in all three brain tissue fractions, with a stronger immunoreactive band in the SD fraction representative of the non-synaptosomal compartment (Fig. 2B). The endogenous mouse apoE levels varied between brain regions with the highest levels found in the hippocampus (Fig. 2C) and the lowest in the thalamus (hippocampus > cerebellum > cortex > thalamus) (*p* < 0.0001, analysis of variance). The endogenous mouse brain apoE levels differed between the mice with a humanized *APOE* ε4/ε4 versus an *APOE* ε2/ε3 liver. Specifically, in the cortex of the mice with *APOE* ε4/ε4 livers (*n* = 8 mice), endogenous mouse apoE levels were lower compared to those found in mice with *APOE* ε2/ε3 livers (*n* = 4 mice) (Fig. 2D). A similar trend was noted in the corresponding fraction from the hippocampi of *APOE* ε4/ε4 liver mice (Fig. 2D). Similarly, there was also a liver *APOE*-genotype-dependent effect on the brain apoE levels in the *APOE* ε4 versus *APOE* ε3 TR mice (Supplementary Fig. 1). Interestingly, the cerebellar SD fraction content of apoE appeared higher in *APOE* ε4/ε4 than ε2/ε3 liver mice (Fig. 2D) whereas no liver *APOE* genotype-dependent effects on the apoE levels were observed in the thalamus (Fig. 2D).

### Altered regional levels of synaptic markers in the brains of *APOE* ε4 humanized-liver mice

**Fig. 3:**
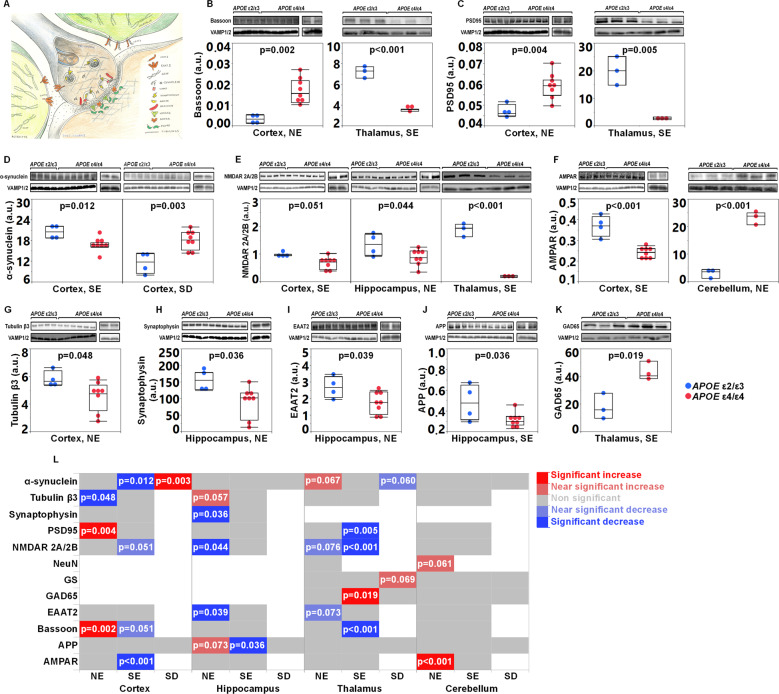
Altered regional levels of synaptic markers in the brains of *APOE* ε4 humanized-liver mice. **A** Graphic illustration of the topological connection between synaptic, neuronal and glial markers assessed in the study. Illustration by Dr Kalicharan Patra. Levels of bassoon (**B**) and PSD95 (**C**) in the cortical NE and thalamic SE fractions of *APOE* ε4/ε4 versus *APOE* ε2/ε3 humanized-liver FRGN mice. **D** α-synuclein levels in the SE and SD fractions isolated from the cortices of *APOE* ε4/ε4 humanized-liver mice. **E** Cortical (SE), hippocampal (NE) and thalamic (SE) levels of NMDAR 2A/2B in FRGN mice with *APOE* ε4/ε4 versus *APOE* ε2/ε3 humanized-livers. **F** AMPAR levels in the SE and NE fractions obtained from the cortex and cerebellum of *APOE* ε4/ε4 and *APOE* ε2/ε3 liver FRGN mice. **G** Levels of tubulin β3 in the cortical NE fraction of *APOE* ε4/ε4 FRGN humanized-liver mice. Hippocampal levels of the synaptic markers synaptophysin (**H**), EAAT2 (**I**) in the NE fraction, and APP (**J**) in the SE fraction as assessed by densitometric analysis of Western blot in the FRGN mice with *APOE* ε4/ε4 versus *APOE* ε2/ε3 livers. **K** Levels of GAD65 in the thalamic SE fraction of FRGN mice with humanized *APOE* ε4/ε4 versus *APOE* ε2/ε3 livers. **L** Heatmap illustrating the overall effects of a liver *APOE*ε4 genotype on the levels of synaptic and neuronal makers assessed in the tissue fractions obtained from the cortex, hippocampus, cerebellum and thalamus of the humanized FRGN liver mice. White panels correspond to proteins that were not assessed in the specific tissue fraction. Densitometric values of Western blot-generated bands are presented as mean or median (minimum–maximum), after undergone normalization against synaptobrevin isoforms 1 and 2. Statistical significance was assessed using the Student’s *t* test except for the group comparison of the NMDAR 2A/2B levels in the cortical SE fraction in which significance was assessed using Wilcoxon signed-rank test. See also Supplementary Fig. 2 and Supplementary Table 4.

Next, the impact of the liver *APOE* genotype on synaptic integrity in various brain regions was assessed. Figure 3A outlines the topographical location of the investigated markers. We focused on the cortex and the hippocampus of the *APOE* ε2/ε3 (*n* = 4) and *APOE* ε4/ε4 (*n* = 8, four from each donor) mice but also investigated the cerebellum and thalamus in a subset of the animals (*APOE* ε2/ε3 (*n* = 3 mice) and *APOE* ε4/ε4 (*n* = 3 mice)). A summary of the assessed synaptic and neuronal markers in the different tissue fractions is described in the Supplementary Table 3 and Fig. 3A.

Comparing the nuclei-enriched (NE) fractions obtained from the cortices from *APOE* ε4/ε4 and *APOE* ε2/ε3 liver mice we detected higher levels of the pre-synaptic marker bassoon (Fig. 3B), the post-synaptic density protein 95 (PSD95) (Fig. 3C), and lower levels of the neuronal microtubule marker tubulin β3 (Fig. 3G). Levels of bassoon and PSD95 were similarly altered in the corresponding fractions and brain region of *APOE* ε4 TR as compared to *APOE* ε3 TR mice (Supplementary Fig. 2A, B). Also, levels of the post-synaptic glutamatergic receptors N-methyl-D-aspartate receptor (NMDAR) 2A/2B and α-amino-3-hydroxy-5-methyl-4-isoxazolepropionic acid receptor (AMPAR) were lower in the cortical SE fraction of mice with livers of the *APOE*ε4 genotype than the *APOE* ε2/ε3 genotype (Fig. 3E, F). In the same fraction, we detected a near-significant 22% decrease in the levels of bassoon in *APOE* ε4/ε4 compared to *APOE* ε2/ε3 FRGN mice (Supplementary Table 4). A similar trend was also observed in the cortical SE fraction of *APOE* ε4 compared to *APOE* ε3 TR mice (0.44 ± 0.25 vs 0.81 ± 0.16 a.u, *p* = 0.091, Student’s *t* test, *n* = 3 mice for each genotype). Mice with an *APOE* ε4/ε4 liver exhibited a shift in the levels of the presynaptic protein α-synuclein from the SE fraction to the SD fraction as the α-synuclein contents were reduced in the synaptosome but increased in the extra-synaptosomal compartment (Fig. 3D). A comparable shift or displacement of α-synuclein from the synaptosomal region to the extra-synaptosomal compartment was also observed in the cortices of *APOE* ε4 versus *APOE* ε3 TR mice (Supplementary Fig. 2C).

Furthermore, in the hippocampi-derived NE fraction of the *APOE* ε4/ε4 liver mice, we observed lower levels of the neuronal glutamatergic marker NMDAR 2A/2B, synaptophysin and the glial glutamate transporter excitatory amino acid transporter 2 (EAAT2), compared to those in NE fraction of the *APOE* ε2/ε3 liver mice (Fig. 3E, H, I). Additionally, in the hippocampal NE fraction of *APOE* ε4/ε4 FRGN mice, tubulin β3 was increased by 14% compared to *APOE* ε2/ε3, however the difference did not reach significance (Supplementary Table 4 outlines findings with p-values ≤0.08). In the same NE fraction we observed 28% higher levels of APP (Supplementary Table 4) in the *APOE* ε4/ε4 liver mice, whereas APP levels in the synaptosomal compartment instead appeared reduced (Fig. 3J). Similar to the observed findings in the humanized-liver mice, protein levels of NMDAR 2A/2B, EAAT2 and APP were lower in the hippocampi of the *APOE* ε4 TR compared to the *APOE* ε3 TR mice (Supplementary Fig. 2D–F). However, there was a significant reduction in the tubulin β3 content in the SE fraction from *APOE* ε4 compared to *APOE* ε3 TR mice (Supplementary Fig. 2G).

In the thalamus, we found an effect of the *APOE* ε4 liver genotype on the synaptosomal protein levels of bassoon, PSD95, NMDAR 2A/2B, as well as the glutamic acid decarboxylase 65-kDa isoform (GAD65) where the latter was increased and the former markers decreased (Fig. 3B, C, E, K). Contrary to the SE fraction, in the thalamic NE and SD fractions, there were only trends, although near statistical significance, towards altered protein levels (Supplementary Table 4). In the cerebellum, a region long considered unaffected in neurodegenerative disorders like AD [37], we detected increased levels of AMPAR (Fig. 3F) and elevated amounts of NeuN in the NE fraction from the *APOE* ε4/ε4 than ε2/ε3 liver mice (Supplementary Table 4). A summary of the findings of assessed synaptic and neuronal markers is illustrated in Fig. 3L and Supplementary Table 4.

### Associations between an *APOE* ε4 liver genotype and markers of insulin signaling in the brain

**Fig. 4:**
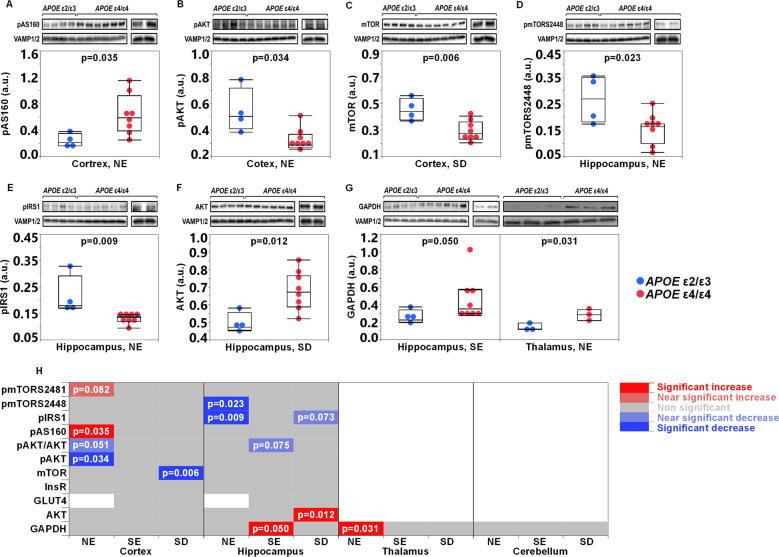
Associations between an *APOE ε4* liver genotype and markers of insulin signaling in the brain. Graphs demonstrating levels pAS160 (**A**) and AKT phosphorylated at serine 473 residue (pAKT) (both in the NE fraction) (**B**), and mTOR in the SD (**C**) fractions obtained from the cortices of *APOE* ε4/ε4 mice compared to *APOE* ε2/ε3. Levels of pmTORS2448 (**D**), pIRS1 (**E**) and AKT (**F**) in the hippocampal NE (**D**, **E**) and SD (**F**) fractions of *APOE* ε4/ε4 versus *APOE* ε2/ε3 humanized-liver mice. **G** Levels of GAPDH in the SE and NE fractions isolated from the hippocampus and thalamus of FRGN mice with humanized *APOE* ε4/ε4 versus *APOE*ε2/ε3 livers. **H** Heatmap showing the effect of an *APOE *ε4 liver genotype on the insulin signaling-related markers in the tissue fractions obtained from the cortex and hippocampus, of the FRGN humanized-liver mice. White panels correspond to proteins that were not assessed in the respective tissue fraction. Marker levels were assessed using western blot and densitometry, the levels were normalized against those of the synaptobrevin isoforms 1 and 2 (VAMP1/2) and the obtained data is presented as mean or median (minimum–maximum). *p*-values were acquired by using the Student’s *t* test (**A**, **C**, **D**, **F**), or the Wilcoxon signed-rank test for the group comparison of GAPDH in the hippocampal SE fraction of *APOE* ε4/ε4 versus *APOE* ε2/ε3 mice. See also Supplementary Figs. 3, 4A and Supplementary Table 4.

As brain insulin resistance can be observed many years before the onset of cognitive symptoms in AD [38], we investigated whether the brains of the mice with humanized *APOE* ε4/ε4 livers exhibited changes in key markers of the insulin signaling pathway in the cortex and hippocampus. In the cortical NE fraction from the *APOE* ε4/ε4 liver mice there were higher levels of the phosphorylated protein designated AKT substrate of 160 kDa (pAS160, phosphorylated at Thr462) (Fig. 4A) and lower levels of phosphorylated (Ser473) AKT (pAKT) than those from the *APOE* ε2/ε3 liver mice (Fig. 4B). Also the pAKT/AKT ratio appeared lower in *APOE* ε4/ε4 mice compared to *APOE* ε2/ε3 mice, however without reaching significance (Supplementary Table 4). In the cortical SD fraction obtained from *APOE* ε4/ε4 humanized-liver mice, we found lower levels of the mammalian target of rapamycin (mTOR) compared to those in *APOE* ε2/ε3 mice (Fig. 4C). In addition, in the cortical NE fraction of *APOE* ε4/ε4 mice, there was a slight increase in the levels of phosphorylated mTOR at serine 2481 (pmTORSer2481) (Supplementary Table 4). No effects on any of the assessed insulin signaling markers could be found in the synaptosomal compartment. In the cortices of *APOE* ε4 TR mice, we found near-significantly lower protein levels of mTOR (*APOE* ε4/ε4 (*n* = 3 mice) average: 0.35 ± 0.08, *APOE* ε3/ε3 (*n* = 3 mice) average: 0.54 ± 0.13, *p* = 0.086, Student’s *t* test).

In the hippocampal NE fraction of *APOE* ε4/ε4 liver mice, there were lower levels of phosphorylated mTOR (pmTORSer2448) and phosphorylated insulin receptor substrate 1 (pIRS1Ser612) (Fig. 4D, E). Both molecules are involved in the terminal steps of the insulin-signaling cascade [39, 40]. In the hippocampi-derived SD fractions, levels of AKT were higher (Fig. 4F) and there was a trend towards a near 60% reduction of pIRS1 (Supplementary Table 4) in *APOE* ε4/ε4 liver mice compared to those in *APOE* ε2/ε3 liver mice. Last, we examined levels of the insulin signaling related protein glyceraldehyde 3-phosphate dehydrogenase (GAPDH) which was shown to be associated with the levels of phosphatidylinositol 4,5-bisphosphate [41] and pAKT [42]. In the SE fraction of the hippocampus of the *APOE* ε4/ε4 liver mice as well as in the NE fraction of thalamus, we observed higher levels of GAPDH than those found in *APOE* ε2/ε3 mice (Fig. 4G). However, in the *APOE* ε4 TR mice there was a decrease in the expression of GAPDH (Supplementary Fig. 4A). In the hippocampal SE fraction obtained from *APOE* ε4/ε4 FRGN mice, the pAKT/AKT appeared lower compared to that in *APOE* ε2/ε3 mice (Supplementary Table 4). Key components in the insulin-signaling pathway are illustrated in Supplementary Fig. 3 and a summary of the assessed insulin signaling-related markers is given in Fig. 4H and Supplementary Table 4.

### Brain tissue levels of neuroinflammation markers

**Fig. 5:**
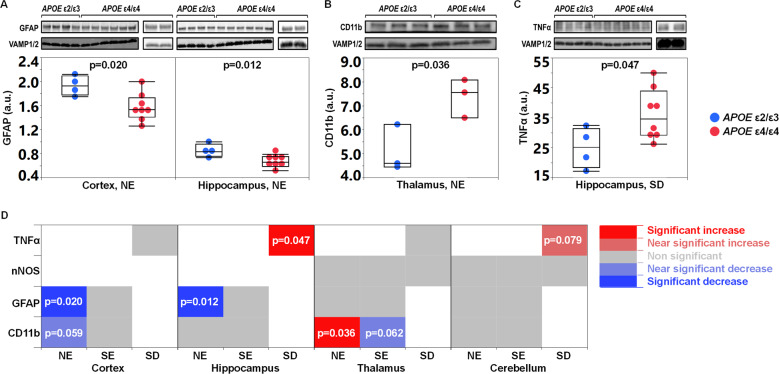
Brain tissue levels of astrocyte and microglia markers and the pro-inflammatory cytokine TNFα. **A** Levels of GFAP in the NE fractions isolated from the cortex and hippocampus of *APOE* ε2/ε3 versus *APOE* ε4/ε4 FRGN humanized-liver mice. **B** CD11b levels in the thalamic NE fraction of *APOE* ε2/ε3 versus *APOE* ε4/ε4 FRGN humanized-liver mice. **C** TNFα levels in the hippocampal SD fraction of humanized *APOE* ε2/ε3 versus *APOE* ε4/ε4 liver FRGN mice. **D** Heatmap demonstrating the overall effects of an *APOE* ε4/ε4 versus an *APOE* ε2/ε3 humanized-liver on the expression of glial markers (GFAP-astrocytes, CD11b-microglia) and the pro-inflammatory cytokine TNFα, in the fractions obtained from the cortex, hippocampus, cerebellum and thalamus of the FRGN humanized-liver mice. White panels correspond to proteins that were not assessed in the specific tissue fraction. Marker levels were assessed using densitometric analysis of immunoreactive western blot bands and normalization against the synaptobrevin isoforms 1 and 2 (VAMP1/2) and the obtained data is represented as mean or median (minimum–maximum). *p*-values were generated by using the Student’s *t* test. See also Supplementary Fig. 4B, C, as well as Supplementary Table 4.

Neuroinflammation, promoted mainly by activated glial cells like astrocytes and microglia is a prominent feature of AD pathophysiology [43]. The FRGN mouse model is immune-suppressed due to the lack of *Rag* and *Il2rg* which render them deficient in mature T-, B- and natural killer (NK) cells but not in other immune cells like monocytes/macrophages and neutrophils [44, 45]. We assessed potential differences in key neuroinflammatory elements (Supplementary Table 3, Fig. 3A) in their brains (cortex, hippocampus, thalamus and cerebellum). Astrogliosis was assessed by examining the levels of the astrocytic marker glial fibrillary acidic protein (GFAP) and potential microgliosis was assessed by investigating the tissue levels of the microglial marker cluster of differentiation molecule 11b (CD11b). In the NE fraction from the cortex and hippocampus of *APOE* ε4/ε4 FRGN mice, levels of GFAP were lower compared to those in *APOE* ε2/ε3 mice (Fig. 5A). This pattern was also seen in the hippocampal SE fraction of *APOE* ε4 TR mice (Supplementary Fig. 4B). Levels of GFAP were not altered in the thalamus and cerebellum. As for CD11b, in the cortical NE fraction obtained from *APOE* ε4/ε4 liver mice (*n* = 8 mice), there was a near-significant 31% reduction in the levels of CD11b compared to those in *APOE* ε2/ε3 liver mice (*n* = 4 mice) (Supplementary Table 4). Contrary to the cortex, in the NE fraction of the thalamus, CD11b levels were elevated in the *APOE* ε4/ε4 liver mice (Fig. 5B), but there was a trend towards a 39% decrease in the expression of CD11b in the thalamic SE fraction of the same mice (Supplementary Table 4). Lower levels of CD11b were also observed in the SE fraction of the hippocampus of *APOE* ε4 TR mice (Supplementary Fig. 4C). No *APOE* liver genotype-dependent alterations in CD11b levels were found in the hippocampus and cerebellum of FRGN mice. There were higher levels of the pro-inflammatory cytokine tumor necrosis factor alpha (TNFα) in the SD fraction obtained from the hippocampus of *APOE* ε4/ε4 FRGN mice (Fig. 5C). A similar *APOE* liver genotype-dependent effect on the levels of TNFα in the cerebellar SD fraction of *APOE* ε4/ε4 mice was observed (Supplementary Table 4). No changes in TNFα levels were found in the *APOE* TR mice. A summary of the assessed neuroinflammation-related markers is given in Fig. 5D and Supplementary Table 4.

### Plasma levels of apoE4 levels are associated with levels of brain apoE, and markers of insulin signaling and synaptic integrity in the cortex and the hippocampus

Since plasma apoE4 levels differed between the *APOE* ε4/ε4 mice transplanted with hepatocytes from two different donors (*p* = 0.023, Wilcoxon signed-rank test), we assessed for potential associations before and after adding ‘donor’ as co-factor in our linear regression model. To ensure that plasma apoE4 levels were not biased by the level of humanization of the FRGN mouse livers we assessed potential correlations between plasma apoE and albumin levels, the latter indicative of humanization/repopulation of the mouse liver with primary human hepatocytes [22]. We found no effect of liver humanization on the levels of human apoE4 (*n* = 10 mice, β (95% CI): 0.67 (−5.02, 6.35), *p* = 0.790) even after adjusting for the *APOE* ε4/ε4 donor (β (95% CI): 1.42 (−4.16, 6.99), *p* = 0.567). Instead, using both regression models higher endogenous mouse apoE levels, specifically in the cortical extra-synaptosomal fraction, were associated with higher plasma human apoE4 levels (Table 1). Plasma human apoE4 levels were related to alterations in the levels of several of the studied brain tissue markers mainly in the hippocampal area (Table 1), with some of them, mainly the markers not directly related to insulin signaling, remaining after using the regression model plasma apoE4*APOE ε4/ε4 donors (Table 1). The observed associations between plasma apoE4 levels and markers of insulin signaling, synaptic integrity and neuroinflammation in the hippocampus were all negative suggesting that higher plasma apoE4 levels are overall disadvantageous for the studied markers in the hippocampal brain region. In the cortex, lower levels of markers of insulin signaling were associated with higher plasma apoE4 levels (Table 1).

**Table 1:** Correlations between the levels of human apoE4 in the plasma of the *APOE* ε4/ε4 humanized-liver mice with brain apoE in the cortical SD fraction as well as with synaptic and insulin signaling related markers in the NE, SE and SD fractions isolated from cortex and hippocampus.

				Model 1: plasma apoE4		Model 2: plasma apoE4*APOE ε4/ε4 donors	
Brain areas	Studied markers	Fraction	Number of samples	Estimates (95% CI)	*p*-value	Estimates (95% CI)	*p-*value
Cortex	apoE	SD	7	8.73 (1.64, 15.8)	0.025	6.26 (2.91, 9.61)	0.007
	InsR	SE	7	−4.27 (−7.99, −0.54)	0.032	−2.74 (−5.38, −0.09)	0.045
	pAS160	SE	7	−2.98 (−5.46, −0.512)	0.027	−1.92 (−4.10, 0.26)	0.071
	GLUT4	SD	6	−2.68 (−4.08, −1.28)	0.006	−0.87 (−3.88, 2.14)	0.426
	pIRS1	SD	7	−1.34 (−2.55, −0.13)	0.036	−0.56 (−3.42, −2.29)	0.613
Hippocampus	AMPAR	SE	7	−2.45 (−3.67, −1.24)	0.005	−2.19 (−4.14, −0.24)	0.036
	Bassoon	SE	7	−0.72 (−1.18, −0.26)	0.016	−0.58 (−1.42, 0.27)	0.131
	NMDAR 2A/2B	SE	7	−6.16 (−8.56, −3.75)	0.004	−4.57 (−6.98, −2.16)	0.009
	PSD95	SE	7	−1.90 (−2.25, −1.56)	<0.001	−1.50 (−3.16, 0.17)	0.067
	Tubulin β3	SE	7	−5.48 (−10.2, −0.79)	0.034	−1.49 (−2.65, −0.33)	0.023
	APP	SD	7	−2.16 (−3.80, 0.52)	0.022	−2.25 (−3.65, −0.85)	0.011
	α-synuclein	SD	7	−3.50 (−5.15, −1.85)	0.003	−2.98 (−6.26, 0.30)	0.065
	pmTORS2481	NE	7	−2.91 (−5.15, 0.66)	0.021	2.74 (−4.83, 10.3)	0.372
	pAKT	NE	7	−1.74 (−3.30, −0.17)	0.036	−0.85 (−2.87, 1.17)	0.309
	mTOR	SE	7	−1.47 (−2.61, −0.34)	0.020	−0.83 (−2.60, 0.94)	0.262
	pAS160	SE	7	−2.58 (−4.23, −0.93)	0.012	−1.48 (−3.62, 0.67)	0.129
	GLUT4	SD	7	−1.37 (2.31, −0.43)	0.015	−1.03 (−2.60, 0.53)	0.141
	pAKT	SD	7	−2.31 (−4.51, −0.10)	0.044	−1.21 (−4.72, 2.30)	0.394
	GAPDH	SD	7	−3.65 (−6.14, −1.16)	0.013	−2.48 (−5.50, 0.54)	0.085

Estimates are shown with 95% confdence interval (CI) as unadjusted (Model 1) and adjusted with *APOE* ε4/ε4 donor as a co-factor (Model 2).

*NE* Nuclei enriched fraction, *SE* Synaptosomal enriched fraction, *SD* Synaptosomal depleted fraction, *InsR* insulin receptor (b-subunit), *pAS160* phospho (Thr462) AKT substrate of 160 kDa, *GLUT4* glucose transporter 4, *pIRS1* phospho (Ser612)-insulin receptor stubstrate 1, *AMPAR* α-amino-3-hydroxy-5-methyl-4-isoxazolepropionic acid receptor, *NMDAR 2A/2B* N-methyl-D-aspartate receptor 2A/2B, *PSD95* post synaptic density 95, *APP* amyloid precursor protein, *pmTORS2481* phospho (Ser2481) mTOR, *pAKT* phospho (Ser473)-protein kinase B, *mTOR* mammalian target of rapamycin, *GAPDH* glyceraldehyde 3-phosphate dehydrogenase.

## Discussion

Recent studies support a role for the liver in the pathophysiology of neurodegenerative diseases. For example, C57BL/6J mice synthesizing human amyloid-β in the liver (hepatocyte-specific human amyloid (HSHA) strain) exhibited an AD-like neurodegenerative phenotype [46] and targeting specifically the liver-brain axis and lipid metabolism using Hop-derived flavonoids improved cognition in mice fed a high-fat diet [47]. Furthermore, the livers of AD patients exhibit altered levels of amyloid-β degrading enzymes [48] and Bassendine and colleagues speculated that AD is a liver-disease of the brain [49]. In support, altered bile acid profiles, products of the liver and the gut microbiome, were associated with AD fluid and imaging biomarkers in patients with mild cognitive impairment (MCI) and AD [50]. The serum-based markers of liver function aspartate aminotransferase (AST) and alanine aminotransferase (ALT) and the ratio thereof correlated with an AD diagnosis, cognition, AD biomarkers and brain glucose metabolism in a large sample of participants of the AD Neuroimaging Initiative, and may therefore offer novel diagnostic and therapeutic opportunities [51].

Our results demonstrate alterations in brain parenchymal levels of mouse endogenous apoE and changes in the protein levels of synaptic glutamate receptors, the pre-synaptic protein α-synuclein as well as molecules involved in brain insulin signaling, promoted by a hepatic *APOE* ε4 genotype. Furthermore, plasma apoE levels in the mice with the humanized *APOE* ε4/ε4 livers were linked to changes in various marker levels that together could be perceived as pathological changes in the brain, *i.e*., higher plasma apoE4 levels were associated with an overall negative outcome. These results provide a first proof-of-concept of a direct link between the *APOE* ε4 genotype of the liver and pathological changes often occurring in the brain during age-related cognitive decline, cognitive injury following environmental challenges and neurodegenerative diseases like AD. Our results also support the notion that plasma apoE does not cross the blood-brain-barrier [19] but instead may act as a facilitator or marker of a liver-related *APOE* ε4 phenotype promoting brain injury and neurodegeneration.

Our results suggest that in addition to a shift from mouse endogenous α-synuclein from the synaptosomal to the extra-synaptosomal compartment in the cortex, mouse endogenous APP protein levels are reduced in the hippocampal synaptosomal fraction in mice with humanized *APOE* ε4 livers. A slight increase in APP levels, although not statistically significant, was instead observed in the hippocampal NE fraction. Hence, although the FRGN humanized-liver mice do not express human versions of α-synuclein and APP, our results indicate that a hepatic *APOE* ε4 genotype may affect the levels of these two key neurodegeneration-related proteins.

The presence of a humanized-liver with the human *APOE* ε4 genotype affected the central nervous system endogenous mouse apoE levels. A relationship between the *APOE* ε4 genotype and lower levels of brain apoE has been documented in mice [52] and humans [52]. Although FRGN mice with humanized-livers and the *APOE* TR mouse models differ in their production of human apoE, with FRGN humanized-liver mice expressing human apoE only in the liver, we detected a similar *APOE* genotype-dependent decrease in the levels of brain apoE both in the cortex and hippocampus of the FRGN humanized *APOE* ε4 liver mice, and in the cortex of *APOE* ε4 TR mice. Whether the observed reduced levels of apoE is related to a reduction in astrocytes, which has been described to occur in older AD patients [53], is not clear. Recently it was shown that reduced levels of pre-synaptic hippocampal apoE may promote cognitive resilience in AD patients [54] hence, local variations in apoE levels in defined brain areas may play an important role in clinical symptomatology.

Lack of apoE in mice was previously shown to create hypercholesterolemia [55] and restoring plasma apoE levels could improve cognitive functions and partially alleviated synaptic deficits in apoE deficient mice. Thus, both plasma and central levels of apoE may independently affect brain health [56]. Intriguingly, Huynh and colleagues suggested that a specific deletion of liver-generated apoE leading to lower plasma apoE levels did not affect brain amyloid-β pathology [25] hence human hepatic apoE plasma levels may not solely affect neurodegenerative processes in the brain but function as a surrogate marker of processes driven by an *APOE* ε4 liver phenotype potentially including phenotypical changes affecting more than just the apoE levels. Our results support the notion that potential *APOE*-directed therapeutic strategies should not include means to increase the levels of plasma apoE4 [20], which consistently have been shown to be lower in *APOE* ε4-carriers [16, 21, 57] since higher plasma apoE4 levels in the FRGN humanized-liver mice were linked to negative outcomes in the brain tissues. These data are consistent with a dominant negative effect of plasma apoE4 rather than reduced beneficial effects due to reduced apoE levels, as supported by comparing apoE deficient mice expressing apoE4 in brain with those expressing no apoE at all [58, 59].

In addition to major effects on the cortex and hippocampus, recent studies have also highlighted the thalamus [60] and the cerebellum [61] as vulnerable brain areas in AD. A study by Cacciaglia and colleagues demonstrated a dose-dependent effect of the *APOE* ε4 allele οn thalamic gray matter volume in cognitively healthy individuals [62]. A positive link between a larger gray matter volume and microglia activation was also documented in mild cognitive impairment (MCI) patients regardless of amyloid-β pathology [63]. Apart from higher tissue levels of CD11b indicating microglia activation in our study, we also found that a liver *APOE* ε4 genotype altered the synaptic integrity also in the thalamus but to a lesser degree in the cerebellum.

Many studies have previously documented a detrimental effect of the *APOE* ε4 genotype on synaptic plasticity [64, 65], glucose hypometabolism [66] and insulin resistance [7]. However, our study is to our knowledge the first to associate these pathological changes in the brain to the presence of a humanized *APOE* ε4/ε4 liver in mice. The liver might play a yet under-appreciated role in age-related cognitive decline, brain injury following environmental challenges, and in the pathogenesis of neurodegenerative diseases like AD. Our hypothesis is supported by the data showing alterations in markers that are key players in various pathophysiological events linked to neurodegenerative diseases like AD. The changes observed in markers linked to the insulin signaling cascade (pAKT, AKT, pAS160, mTOR and pmTORS2448) suggest an association between a liver *APOE* ε4 genotype and the brain PI3K/AKT/mTOR pathway involved in cellular glucose uptake through translocation of glucose transporter 4 (GLUT4) to the plasma membrane [67]. Previous studies have shown an association between *APOE* ε4 and lower levels of pAKT in humans [8] and in *APOE* ε4 TR mice [68, 69]. Reduced glucose metabolism in parietal, temporal and posterior cingulate regions, as assessed with FDG-PET, was previously linked to *APOE* ε4 in non-demented subjects, and in subjects at risk of AD [9, 70, 71]. We have also earlier reported that a higher ratio of plasma apoE4 to apoE3 in cognitively healthy *APOE* ε3/ε4 subjects was linked to reduced glucose metabolism specifically in the hippocampus [20]. This finding could in part be explained by a specific correlation between plasma apoE3 (and not plasma apoE4) and plasma glucose levels where low plasma apoE3 levels were correlated with higher plasma glucose. Higher plasma glucose levels in turn were related to a lower cerebral metabolic rate for glucose CMRgl [72]. Taken together, altered glucose metabolism, insulin resistance and *APOE* ε4 genotype seem to interact and promote an AD-like phenotype, especially in the hippocampus [13].

Shortcomings in our study include the small mouse sample size and the inability to assess gender-dependent effects, as well as a very limited number of hepatocyte donors. However, the absence of significant differences in brain marker levels in mice generated by use of hepatocytes from two different *APOE* ε4/ε4 donors, enhance our hypothesis of an overall effect of *APOE* ε4 genotype on brain integrity. As the frequency of *APOE* ε2 and ε4 homozygosity is rare (less than 1% for ε2 and less than 4% for ε4 http://www.alzgene.org/meta.asp?geneID=83) acquisition of primary human hepatocytes from donors with these genotypes is very difficult. Furthermore, not all primary human hepatocyte cultures successfully repopulate the rodent liver. Our study is to our knowledge the first to report brain-specific experimental data from FRGN humanized liver mice with different *APOE* liver genotypes. Future studies are warranted to further develop this humanized liver mouse model potentially also including hepatocyte ex-vivo gene editing [73] and to establish whether our observations are due to the presence or merely the absence of *APOE* ε4 in the FRGN mice with humanized *APOE* ε2/ε3 livers. Importantly, it needs to be elucidate whether the herein reported changes in the brain tissues translate into behavioral alterations and cognitive deficits. Causal mechanisms driving *APOE* ε4 pathological changes in the brain via the liver may relate to lipid metabolism, known to be modulated by *APOE* genotype, where in addition specific liver-secreted players in an *APOE* genotype-dependent manner adversely affect the blood-brain-barrier and the cerebrovasculature. These factors may together elicit pathological effects by driving the so called vascular contributions to cognitive impairment and dementia (VCID) [74]. Unraveling the underlying mechanisms may shed crucial new light on the pathogenesis of neurodegenerative diseases like AD and facilitate the development of novel therapeutic strategies where the liver and liver-promoted processes may be targeted.

## Supplementary information

supplementary information
