# Supplementary material for: Brain integrity is altered by hepatic APOE ε4 in humanized-liver mice
Source: Mol Psychiatry. 2022 Apr 13;27(8):3533–43. doi: 10.1038/s41380-022-01548-0 (PMC9708568; doi:10.1038/s41380-022-01548-0)
Supplement: Supplementary file 1 — supplementary information [file 41380_2022_1548_MOESM1_ESM.docx]

**SUPPLEMENTARY INFORMATION**

**Brain integrity is altered by hepatic *APOE*ε4 in humanized-liver mice**

Andreas Giannisis^1^, Kalicharan Patra^1^, Anna K. Edlund^1^, Lur Agirrezabala Nieto^1^, Joan Benedicto-Gras^1^, Simon Moussaud^1^, Andrés de la Rosa^1^, Daniel Twohig^1^ Tore Bengtsson^2^, Yuan Fu^3^, Guojun Bu^3^, Greg Bial^4^, Lander Foquet^4^, Christina Hammarstedt^5^, Stephen Strom^5^, Kristina Kannisto^5^, Jacob Raber^6^, Ewa Ellis^7^ and Henrietta M. Nielsen^1,*^

^*^Correspondence: [henrietta.nielsen@dbb.su.se](mailto:henrietta.nielsen@dbb.su.se)

The supplementary information includes detailed Materials and Methods, four tables (Supplementary Table 1-4) and four figures (Supplementary Figure 1-4)

**Materials and Methods**

*In vivo models*

Mice were originally obtained from Yecuris Corporation (Tualatin, OR, USA), and maintained in breeding colonies at the Karolinska Institutet Animal Facility. Human hepatocytes were isolated from liver tissues derived from donors (Supplementary Table 1) in line with a previously published protocol ^1^ and in agreement with the local ethical permit (2017/269-31). A suspension of one million viable hepatocytes was injected into the spleen parenchyma of mice anesthetized with isoflurane (Baxter, Norfolk, UK) when they were 6-8 weeks old. Appropriate analgesia was administered during the time of surgery and 24 hours after the surgical procedure. Mice were throughout the study maintained on the PicoLab High Energy Mouse Diet with 18.9% protein (Animal Specialties and Provisions, Quakertown, PA, USA), and supplemented with NTBC (Nitisinone) to avoid lethal liver damage. After cell transplantation, animals were cycled on-and-off of NTBC to support engraftment and the proliferation of donor-human hepatocytes. Engraftment of human hepatocytes was monitored by assessing the human albumin levels in blood samples collected twice a month through the tail vein. In previously published studies it was estimated that 1 mg/mL of circulating human albumin represents a 20% level of repopulation with human hepatocytes ^2, 3^.

*Brain tissue fractionation*

Each tissue sample was centrifuged at 2000xg for 10 minutes at 4°C and the resulting supernatant (S1) and pellet (P1) were separated. The pellet was re-suspended in 10 volumes HB, re-centrifuged under the same conditions as above. The resulting supernatant was discarded and the pellet re-suspended in lysis buffer (LB) (HB with 1% SDS) constituting the NE fraction. The additional two fractions were obtained from further processing of the S1 fraction. Specifically, S1 was centrifuged at 15000xg for 15 minutes and the resulting supernatant (S2) was transferred to a new tube along with 1% SDS yielding the SE fraction. Last, the pellet (P2) was re-suspended in 10 volumes of HB, re-centrifuged and the obtained supernatant was discarded. The pellet was lysed in LB to yield the SE fraction. All fractions were sonicated (15 sec ON-10 sec OFF-15 sec ON-STOP at 30% amplitude), boiled at 70^°^C for 10 minutes and total protein concentrations were determined using the bicinchoninic acid (BCA) protein assay (Thermo Fischer) where after the fractions were stored at -20^°^C until analyzed.

*Quantification of plasma apoE levels*

In brief, a 96 well plate was coated with a mouse monoclonal pan-apoE antibody, WUE4 (final concentration 1 μg/mL, Novus Biologicals) diluted in 0.05 M sodium carbonate buffer (NaN_3_, NaHCO_3_, Na_2_CO3, pH 9.6) at room temperature overnight. The next day, following washing, blocking (in 1% w/v non-fat dry milk powder in PBS) and sample incubation, captured plasma apoE was detected with the biotinylated goat polyclonal pan-apoE detection antibody (final concentration 0.2 μg/mL, Meridian Lifesciences) and a quantifiable signal produced using HRP-conjugated streptavidin (final concentration 1 μg/mL, Fitzgerald) and tetramethylbenzidine (TMB, Sigma-Aldrich) as the substrate. The enzymatic reaction was stopped by the addition of 1 M H_2_SO_4_ and the optical density was measured at 450 nm using the HiPo-96 microplate photometer (BIOSAN, Riga, Latvia).

| Donor | *APOE* genotype | Gender | Age | Cause of liver resection/explant surgery | Number of transplanted animals |
| --- | --- | --- | --- | --- | --- |
| #1 | ε2/ε3 | Female | 25 | Colorectal cancer | 7 |
| #2 | ε4/ε4 | Female | 60 | Pseudoyxom peritoni | 7 |
| #3 | ε4/ε4 | Male | 25 | Suicide, organ donor | 4 |

**Supplementary Table 1:** Clinical characteristics of the liver donors

| Antibodies | Supplier | Catalogue Number |
| --- | --- | --- |
| Mouse monoclonal anti-bassoon (clone 219E1) | Synaptic Systems | Cat# 141 011  RRID: [AB_2619827](http://antibodyregistry.org/AB_2619827) |
| Rabbit polyclonal anti-GluN 2A/B (N-methyl-D-aspartate receptor (NMDAR 2A/2B)), | Synaptic Systems | Cat# 244 003  RRID: [AB_10804284](http://antibodyregistry.org/AB_10804284) |
| Rabbit polyclonal anti-GluA1-4 (α-amino-3-hydroxy-5-methyl-4-isoxazolepropionic acid receptor (AMPAR)) | Synaptic Systems | Cat# 182 403  RRID: [AB_10598611](http://antibodyregistry.org/AB_10598611) |
| Mouse monoclonal anti-post synaptic density 95 kDa (PSD95), (clone 6G6-1C9) | Novus Biologicals | Cat# NB300-556  RRID: [AB_2092366](http://antibodyregistry.org/AB_2092366) |
| Rabbit monoclonal anti-synaptophysin  (clone SP11) | Thermo Fisher Scientific | Cat# MA5-14532  RRID: [AB_10983675](http://antibodyregistry.org/AB_10983675) |
| Mouse monoclonal anti-synaptobrevin isoforms 1 and 2 (VAMP1/2) (Clone 540524) | R&D Systems | Cat# MAB5958  RRID: [AB_1964742](http://antibodyregistry.org/AB_1964742) |
| Sheep polyclonal anti-α-synuclein | Abcam | Cat# ab6162  RRID: [AB_2192805](http://antibodyregistry.org/AB_2192805) |
| Rabbit polyclonal anti-glutamic acid decarboxylase 65 kDa isoform (GAD65) | Thermo Fisher Scientific | Cat# PA5-22260  RRID: [AB_11154107](http://antibodyregistry.org/AB_11154107) |
| Monoclonal anti-amyloid precursor protein (clone mC99 (70-80)) | Milipore | Cat# MABN380  RRID: [AB_2714163](http://antibodyregistry.org/AB_2714163) |
| Rabbit polyclonal anti-excitatory amino acid transporter 2 (EAAT2) | Synaptic Systems | Cat# 250 203  RRID: [AB_11042312](http://antibodyregistry.org/AB_11042312) |
| Mouse monoclonal anti-Glutamine synthetase (clone GT7711) | Thermo Fisher Scientific | Cat# MA5-27750  RRID: [AB_2735205](http://antibodyregistry.org/AB_2735205) |
| Mouse monoclonal anti-tubulin β3 (clone TU20) | Cell Signaling Technology | Cat# 4466  RRID: [AB_1904176](http://antibodyregistry.org/AB_1904176) |
| Monoclonal anti-NeuN (clone EPR12763) | Abcam | Cat# ab177487  RRID: [AB_2532109](http://antibodyregistry.org/AB_2532109) |
| Rabbit polyclonal anti-glial fibrillary acidic protein (GFAP) | Agilent Dako | Cat# Z0334  RRID: [AB_10013382](http://antibodyregistry.org/AB_10013382) |
| Rabbit polyclonal anti- cluster of differentiation molecule 11b (CD11b) | Novus Biologicals | Cat# NB110-89474SS  RRID: [AB_2265132](http://antibodyregistry.org/AB_2265132) |
| Rabbit monoclonal anti- tumor necrosis factor alpha (TNFα) (clone D2D4) | Cell Signaling Technology | Cat# 11948  RRID: [AB_2687962](http://antibodyregistry.org/AB_2687962) |
| Rabbit polyclonal anti-Neuronal Nitric oxide synthase (nNOS) | Thermo Fisher Scientific | Cat# 61-7000  RRID: [AB_2313734](http://antibodyregistry.org/AB_2313734) |
| Rabbit Polyclonal anti-mammalian target of rapamycin (mTOR) | Cell signaling technology | Cat# 2972  RRID: [AB_330978](http://antibodyregistry.org/AB_330978) |
| Rabbit polyclonal anti-phospho (Ser2481) mTOR (pmTORS2481) | Cell signaling technology | Cat# 2974  RRID: [AB_2262884](http://antibodyregistry.org/AB_2262884) |
| Rabbit polyclonal anti-phospho (Ser2448) mTOR (pmTORS2448) | Cell signaling technology | Cat# 2971  RRID: [AB_330970](http://antibodyregistry.org/AB_330970) |
| Rabbit polyclonal anti-Insulin receptor (b-subunit) (InsR) (clone 4B8) | Cell signaling technology | Cat# 3025  RRID: [AB_2280448](http://antibodyregistry.org/AB_2280448) |
| Rabbit polyclonal anti-phospho (Ser612)-insulin receptor stubstrate 1 (pIRS1) | Thermo Fisher Scientific | Cat# 44-550G  RRID: [AB_2533678](http://antibodyregistry.org/AB_2533678) |
| Rabbit polyclonal anti-phospho (Thr462) AKT substrate of 160 kDa (pAS160) | Thermo Fisher Scientific | Cat# 44-1071G  RRID: [AB_2533564](http://antibodyregistry.org/AB_2533564) |
| Rabbit polyclonal anti-protein kinase B (AKT) | Cell signaling technology | Cat# 9272  RRID: [AB_329827](http://antibodyregistry.org/AB_329827) |
| Rabbit polyclonal anti-phospho (Ser473)-protein kinase B (pAKT) | Cell signaling technology | Cat# 9271  RRID: [AB_329825](http://antibodyregistry.org/AB_329825) |
| Mouse polyclonal anti-glucose transporter 4 (GLUT4) (clone 1F8) | Cell signaling technology | Cat# 2213  RRID: [AB_823508](http://antibodyregistry.org/AB_823508) |
| Rabbit polyclonal anti-glyceraldehyde 3-phosphate dehydrogenase (GAPDH) | Thermo Fisher Scientific | Cat# PA1-987  RRID: [AB_2107311](http://antibodyregistry.org/AB_2107311) |
| Mouse monoclonal anti-apolipoprotein E  (clone E6D7) | Thermo Fisher Scientific | Cat# MA1-91063  RRID: [AB_1954868](http://antibodyregistry.org/AB_1954868) |
| Mouse monoclonal anti-apolipoprotein E  (clone WUE-4) | Novus Biologicals | Cat# NB110-60531  RRID: [AB_920623](http://antibodyregistry.org/AB_920623) |
| Mouse monoclonal anti-Lamin B1  (clone L-5) | Thermo Fisher Scientific | Cat# 33-2000  RRID: [AB_2533106](http://antibodyregistry.org/AB_2533106) |

**Supplementary Table 2:** Antibodies used in this study for the immunodetection of the studied proteins.

|  | Markers | Fractions | | | Brain areas and mouse model | | |
| --- | --- | --- | --- | --- | --- | --- | --- |
|  |  | **NE** | **SE** | **SD** | | **Mouse models** | **Brain areas** |
| Synaptic markers | Bassoon | x | x |  | | FRGN and TR | Cortex, Hippocampus, Cerebellum & Thalamus |
|  | N-methyl-D-aspartate receptor (NMDAR 2A/2B) | x | x |  | | FRGN and TR | Cortex, Hippocampus, Cerebellum & Thalamus |
|  | α-amino-3-hydroxy-5-methyl-4-isoxazolepropionic acid receptor (AMPAR) | x | x |  | | FRGN and TR | Cortex, Hippocampus, Cerebellum & Thalamus |
|  | post synaptic density  (PSD95) | x | x |  | | FRGN and TR | Cortex, Hippocampus, Cerebellum & Thalamus |
|  | Synaptophysin | x | x |  | | FRGN and TR | Cortex, Hippocampus |
|  | synaptobrevin isoforms 1 and 2 (VAMP1/2) | x | x | x | | FRGN and TR | Cortex, Hippocampus, Cerebellum & Thalamus |
|  | α-synuclein | x | x | x | | FRGN and TR | Cortex, Hippocampus, Cerebellum & Thalamus |
|  | glutamic acid decarboxylase 65-kDa isoform (GAD65) | x | x |  | | FRGN | Cerebellum & Thalamus |
|  | amyloid precursor protein (APP) | x | x | x | | FRGN and TR | Cortex, Hippocampus, Cerebellum & Thalamus |
|  | excitatory amino acid transporter 2 (EAAT2) | x | x |  | | FRGN and TR | Cortex, Hippocampus, Cerebellum & Thalamus |
|  | Glutamine synthetase | x | x | x | | FRGN | Cerebellum & Thalamus |
| Neuronal markers | tubulin β3 | x | x |  | | FRGN and TR | Cortex, Hippocampus, Cerebellum & Thalamus |
|  | NeuN | x |  |  | | FRGN | Cerebellum & Thalamus |
| Glial markers | glial fibrillary acidic protein (GFAP) | x | x | x | | FRGN and TR | Cortex, Hippocampus, Cerebellum & Thalamus |
|  | cluster of differentiation molecule 11b (CD11b) | x | x | x | | FRGN and TR | Cortex, Hippocampus, Cerebellum & Thalamus |
| Inflammatory markers | tumor necrosis factor-alpha (TNFα) |  |  | x | | FRGN and TR | Cortex, Hippocampus, Cerebellum & Thalamus |
|  | neuronal Nitric oxide synthase (nNOS) | x | x | x | | FRGN | Cerebellum & Thalamus |
| Insulin signaling related markers | mammalian target of rapamycin (mTOR) | x | x | x | | FRGN, TR* | Cortex, Hippocampus |
|  | phospho (Ser2481) mTOR  (pmTORS2481) | x | x | x | | FRGN | Cortex, Hippocampus |
|  | phospho (Ser2448) mTOR  (pmTORS2448) | x | x | x | | FRGN, TR* | Cortex, Hippocampus |
|  | Insulin receptor (b-subunit) (InsR) | x | x | x | | FRGN | Cortex, Hippocampus |
|  | phospho (Ser612)-insulin receptor stubstrate 1 (pIRS1) | x | x | x | | FRGN, TR* | Cortex, Hippocampus |
|  | phospho (Thr462) AKT substrate of 160 kDa (pAS160) | x | x | x | | FRGN, TR* | Cortex, Hippocampus |
|  | protein kinase B (AKT) | x | x | x | | FRGN, TR* | Cortex, Hippocampus |
|  | phoshpo (Ser473)-protein kinase B  (pAKT) | x | x | x | | FRGN, TR* | Cortex, Hippocampus |
|  | glucose transporter 4 (GLUT4) | x | x | x | | FRGN | Cortex, Hippocampus |
|  | glyceraldehyde 3-phosphate dehydrogenase (GAPDH) | x | x | x | | FRGN and TR | Cortex, Hippocampus, Cerebellum & Thalamus |
| Apolipoprotein E | mouse apolipoprotein E |  |  | x | | FRGN | Cortex, Hippocampus, Cerebellum & Thalamus |
|  | human apolipoprotein E |  |  | x | | TR | Cortex, Hippocampus |

**Supplementary Table 3:** Markers analyzed by western blot analysis of respective fractions isolated from cortex, hippocampus, cerebellum and thalamus derived from FRGN humanized-liver and *APOE* TR mouse brains. The brain tissue fractions are labeled as NE, SE and SD. NE: Nuclei enriched fraction; SE: Synaptosomal enriched fraction; SD: Synaptosomal depleted fraction. *Proteins assessed only in specific brain areas and fractions of TR mice; pAS160 (Cortex, NE fraction), AKT (Cortex, NE fraction), pAKT (Cortex NE fraction), mTOR (Cortex, SD fraction), pmTORS2448 (Hippocampus, NE fraction), pIRS1 (Hippocampus, NE fraction), AKT (Hippocampus, SD fraction).

| Synaptic markers | Markers | Area / Fraction | *APOE* ε2/ε3 | *APOE* ε4/ε4 | *p-*value | Statistical test |
| --- | --- | --- | --- | --- | --- | --- |
|  | Bassoon | Cortex / SE | 0.79  (0.67 – 1.53) | 0.62  (0.32 – 0.96) | 0.051 | Wilcoxon signed-rank test |
|  | α-synuclein | Thalamus / NE | 10.7 ± 1.55 | 18.8 ± 5.37 | 0.067 | Student’s *t*-test |
|  |  | Thalamus / SD | 18.9 ± 5.97 | 9.46 ± 1.89 | 0.060 | Student’s *t*-test |
|  | NMDAR 2A/2B | Thalamus / NE | 3.47 ± 0.88 | 2.14 ± 0.38 | 0.076 | Student’s *t*-test |
|  | Tubulin β3 | Hippocampus / NE | 1.69 ± 0.22 | 1.93 ± 0.17 | 0.057 | Student’s *t*-test |
|  | EAAT2 | Thalamus / NE | 33.0 ± 9.21 | 14.6 ± 9.47 | 0.073 | Student’s *t*-test |
|  | APP | Hippocampus / NE | 105 ± 16.0 | 135 ± 26.8 | 0.073 | Student’s *t*-test |
|  | NeuN | Cerebellum / NE | 10.7 ± 1.69 | 16.7 ± 3.70 | 0.061 | Student’s *t*-test |
|  | GS | Thalamus / SD | 10.9 ± 1.71 | 20.9 ± 6.80 | 0.069 | Student’s *t*-test |
| Insulin signaling related markers | pmTORS2481 | Cortex / NE | 0.23 ± 0.07 | 0.38 ± 0.15 | 0.082 | Student’s *t*-test |
|  | pIRS1 | Hippocampus / SD | 0.40 ± 0.31 | 0.17 ± 0.09 | 0.073 | Student’s *t*-test |
|  | pAKT/AKT | Cortex / NE | 0.89  (0.82 – 1.77) | 0.59  (0.38 – 1.01) | 0.051 | Wilcoxon signed-rank test |
|  |  | Hippocampus / SE | 0.73  (0.66 – 2.06) | 0.62  (0.46 – 0.83) | 0.075 | Wilcoxon signed-rank test |
| Glial and neuroinflammation markers | CD11b | Cortex / NE | 37.9 ± 2.9 | 26.3 ± 10.4 | 0.059 | Student’s *t*-test |
|  |  | Thalamus / SE | 17.4 ± 4.56 | 10.6 ± 0.46 | 0.062 | Student’s *t*-test |
|  | TNFα | Cerebellum / SD | 3.10 ± 0.72 | 4.74 ± 0.97 | 0.079 | Student’s *t*-test |

**Supplementary Table 4:** Protein levels exhibiting increasing or decreasing trends in the cortex, hippocampus thalamus and cerebellum of *APOE* ε4/ε4 FRGN humanized-liver mice compared to *APOE* ε2/ε3 mice. Group comparisons for cortex and hippocampus were peformed between *n* = 4 *APOE* ε2/ε3 mice and *n* = 8 *APOE* ε4/ε4, whereas for thalamus and cerebellum *n* = 3 mice for each genotype were utilized. NE: Nuclei enriched fraction; SE: Synaptosomal enriched fraction; SD: Synaptosomal depleted fraction. NMDAR2A/2B: N-methyl-D-aspartate receptor, EAAT2: Excitatory amino acid transporter 2, APP: Amyloid precursor protein, GS: Glutamine synthetase, pmTORS2481: phospho (Ser2481) mammalian target of rapamycin, pIRS1: phospho (Ser612)-insulin receptor stubstrate 1, pAKT / AKT: phospho (Ser473) protein kinase B / protein kinase B, CD11b: Cluster of differentiation molecule 11b, TNFα: Tumor necrosis factor-alpha. Protein levels are represented as average ± standard deviation, or median (min-max)


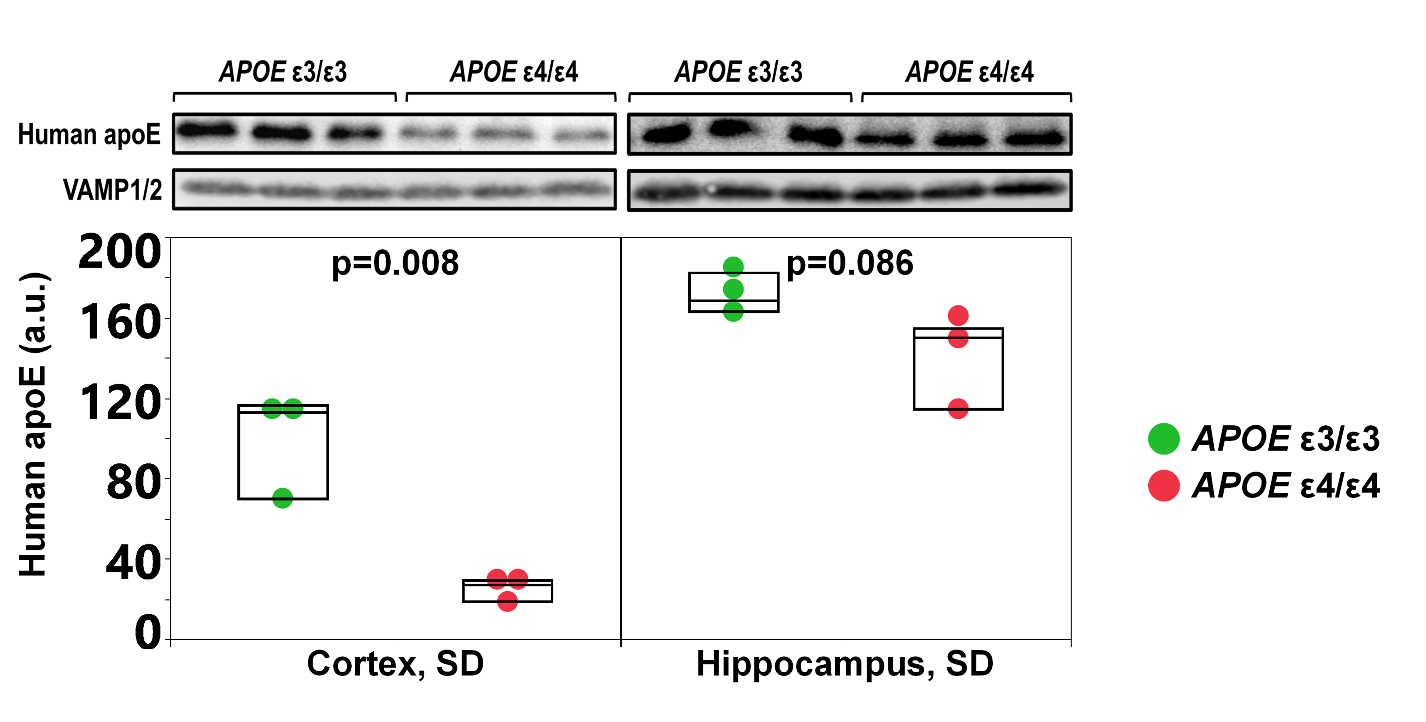


**Supplementary Figure 1:** Effect of the *APOE* ε4 genotype on the brain parenchymal levels of apoE in *APOE* TR mice. Densitometric analysis of apoE antibody reactive bands after normalization against synaptobrevin isoforms 1 and 2 (VAMP1/2) illustrate human apoE levels in the SD fraction obtained from the cortex and hippocampus of *APOE* ε3 versus *APOE* ε4 TR mice. Data is presented as mean or median (minimum – maximum). *p*-values were acquired using the student’s *t*-test.


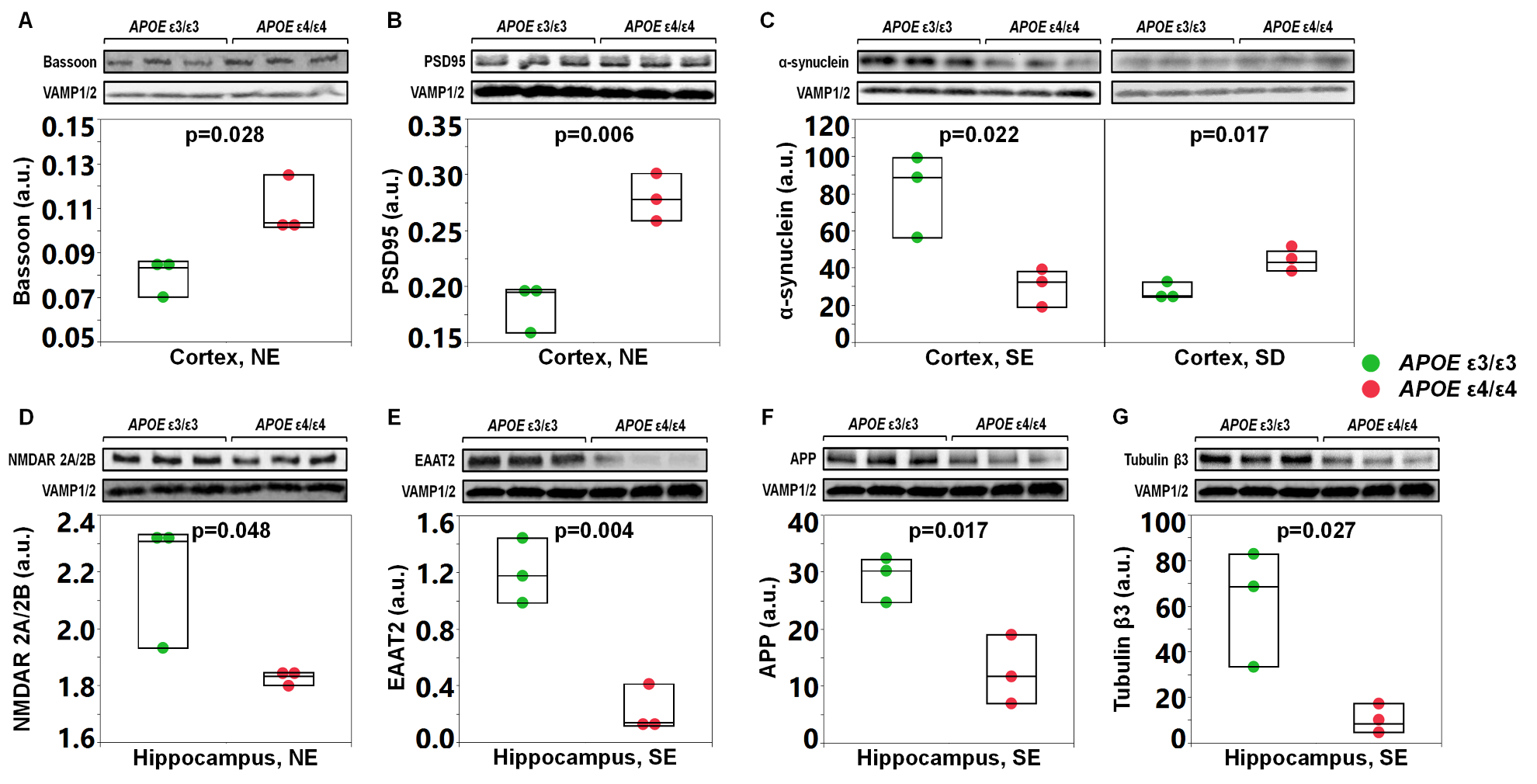


**Supplementary Figure 2:** *APOE* ε4 genotype alters the levels of synaptic proteins in the cortex (A-C) and hippocampus (D-G) of *APOE* TR mice.

(A-B) Levels of bassoon (A) and PSD95 (B) in the NE fraction generated from the cortex of *APOE* ε4 TR compared to *APOE* ε3 TR mice.

(C) Levels of α-synuclein in the cortical SE and SD fractions of *APOE* ε4 versus *APOE* ε3 TR mice.

(D-G) Graphs show protein levels of NMDAR 2A/2B (D), EAAT2 (E), APP (F) and tubulin β3 (G) in the hippocampal NE (D) and SE (E, F, G) fractions of *APOE* ε4 versus *APOE* ε3 TR mice.

Marker levels were assessed using densitometric analysis of immunoreactive western blot bands which were normalization against the synaptobrevin isoforms 1 and 2 (VAMP1/2). Results are represented as mean or median (minimum – maximum). *p*-values were generated by using student’s *t*-test.


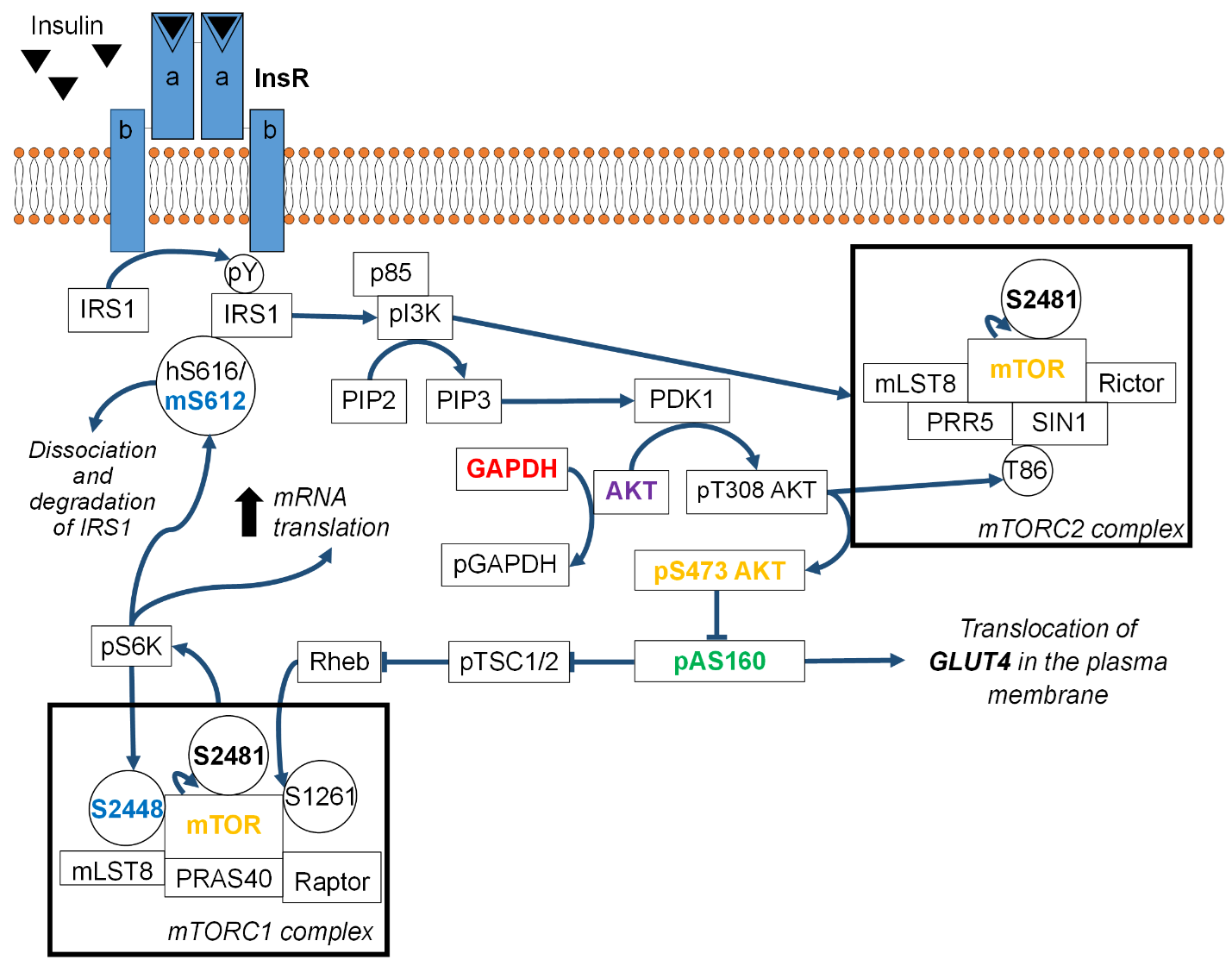


**Supplementary Figure 3:** Illustration of the principal components in the insulin signaling pathway. Binding of insulin to the insulin receptor activates the PI3K/AKT/mTOR pathway and impacts the activity of GAPDH and mTOR complexes 1 and 2 (mTORC1, mTORC2) ^4-11^. Among the different markers that were studied in the current study (showing in bold), presence of the *APOΕ*ε4 genotype in the liver induced an *increase* (green) and a *decrease* in the cortex (orange); an *increase* (purple) and a *decrease* in the hippocampus (blue); an *increase* in the hippocampus and thalamus in FRGN humanized-liver mice (red).


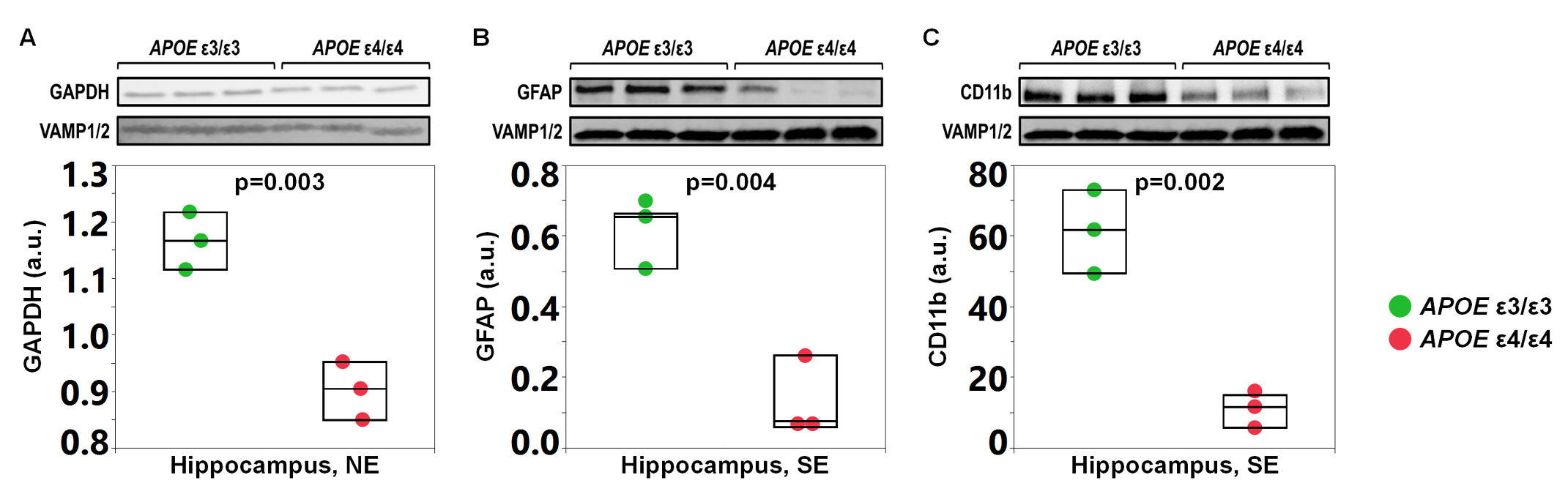


**Supplementary Figure 4:** The *APOE* ε4 genotype alters the GAPDH and glial marker levels in the hippocampus of *APOE* TR mice. Bar graphs represent the densitometric analysis of the western blot immunoreactive bands after normalization against synaptobrevin isoforms 1 and 2 (VAMP1/2) and show the levels of GAPDH (A), GFAP (B) and CD11b (C) in the NE (A) and SE (B, C) fractions of *APOE* ε4 compared to *APOE* ε3 TR mice. Data is represented as mean or median (minimum – maximum) and *p*-values were acquired by use of the student’s *t*-test.

**References**

1. Gramignoli R, Green ML, Tahan V, Dorko K, Skvorak KJ, Marongiu F *et al.* Development and application of purified tissue dissociation enzyme mixtures for human hepatocyte isolation. *Cell Transplant* 2012; **21**(6)**:** 1245-1260.

2. Azuma H, Paulk N, Ranade A, Dorrell C, Al-Dhalimy M, Ellis E *et al.* Robust expansion of human hepatocytes in Fah-/-/Rag2-/-/Il2rg-/- mice. *Nat Biotechnol* 2007; **25**(8)**:** 903-910.

3. Wilson EM, Bial J, Tarlow B, Bial G, Jensen B, Greiner DL *et al.* Extensive double humanization of both liver and hematopoiesis in FRGN mice. *Stem Cell Res* 2014; **13**(3 Pt A)**:** 404-412.

4. Acosta-Jaquez HA, Keller JA, Foster KG, Ekim B, Soliman GA, Feener EP *et al.* Site-specific mTOR phosphorylation promotes mTORC1-mediated signaling and cell growth. *Mol Cell Biol* 2009; **29**(15)**:** 4308-4324.

5. Chang L, Chiang SH, Saltiel AR. Insulin signaling and the regulation of glucose transport. *Mol Med* 2004; **10**(7-12)**:** 65-71.

6. Yang G, Murashige DS, Humphrey SJ, James DE. A Positive Feedback Loop between Akt and mTORC2 via SIN1 Phosphorylation. *Cell Rep* 2015; **12**(6)**:** 937-943.

7. Holz MK, Blenis J. Identification of S6 kinase 1 as a novel mammalian target of rapamycin (mTOR)-phosphorylating kinase. *J Biol Chem* 2005; **280**(28)**:** 26089-26093.

8. Copp J, Manning G, Hunter T. TORC-specific phosphorylation of mammalian target of rapamycin (mTOR): phospho-Ser2481 is a marker for intact mTOR signaling complex 2. *Cancer Res* 2009; **69**(5)**:** 1821-1827.

9. Peterson RT, Beal PA, Comb MJ, Schreiber SL. FKBP12-rapamycin-associated protein (FRAP) autophosphorylates at serine 2481 under translationally repressive conditions. *J Biol Chem* 2000; **275**(10)**:** 7416-7423.

10. Yoon MS. The Role of Mammalian Target of Rapamycin (mTOR) in Insulin Signaling. *Nutrients* 2017; **9**(11).

11. Baba T, Kobayashi H, Kawasaki H, Mineki R, Naito H, Ohmori D. Glyceraldehyde-3-phosphate dehydrogenase interacts with phosphorylated Akt resulting from increased blood glucose in rat cardiac muscle. *FEBS Lett* 2010; **584**(13)**:** 2796-2800.
